# Long-distance airborne dispersal of SARS-CoV-2 in COVID-19 wards

**DOI:** 10.1038/s41598-020-76442-2

**Published:** 2020-11-11

**Authors:** Karolina Nissen, Janina Krambrich, Dario Akaberi, Tove Hoffman, Jiaxin Ling, Åke Lundkvist, Lennart Svensson, Erik Salaneck

**Affiliations:** 1 Department of Medical Sciences, Uppsala University, Uppsala, Sweden; 2 Department of Medical Biochemistry and Microbiology, Uppsala University, Uppsala, Sweden; 3 Department of Molecular Medicine and Virology, Linköping University, Linköping, Sweden; 4 Division of Infectious Diseases, Department of Medicine, Karolinska Institute, Solna, Sweden

**Keywords:** Viral infection, Epidemiology, SARS-CoV-2

## Abstract

Evidence suggests that SARS-CoV-2, as well as other coronaviruses, can be dispersed and potentially transmitted by aerosols directly or via ventilation systems. We therefore investigated ventilation openings in one COVID-19 ward and central ducts that expel indoor air from three COVID-19 wards at Uppsala University Hospital, Sweden, during April and May 2020. Swab samples were taken from individual ceiling ventilation openings and surfaces in central ducts. Samples were subsequently subjected to rRT-PCR targeting the N and E genes of SARS-CoV-2. Central ventilation HEPA filters, located several stories above the wards, were removed and portions analyzed in the same manner. In two subsequent samplings, SARS-CoV-2 N and E genes were detected in seven and four out of 19 room vents, respectively. Central ventilation HEPA exhaust filters from the ward were found positive for both genes in three samples. Corresponding filters from two other, adjacent COVID-19 wards were also found positive. Infective ability of the samples was assessed by inoculation of susceptible cell cultures but could not be determined in these experiments. Detection of SARS-CoV-2 in central ventilation systems, distant from patient areas, indicate that virus can be transported long distances and that droplet transmission alone cannot reasonably explain this, especially considering the relatively low air change rates in these wards. Airborne transmission of SARS-CoV-2 must be taken into consideration for preventive measures.

## Introduction

During the coronavirus infectious disease 19 (COVID-19) pandemic, droplet transmission has been considered the most significant transmission route for severe acute respiratory syndrome coronavirus 2 (SARS-CoV-2), although other routes such as aerosol, fecal–oral, and indirect transmission via fomites may contribute to the rapid global dissemination of the virus^1,2^. The relative importance of aerosols versus droplets in the transmission of respiratory infections is difficult to distinguish, since particles of both aerosol and droplet size are generated for example when talking^3,4^. Aerosols are smaller than droplets, traditionally defined as smaller than 5 µm in diameter, and are thought to remain airborne longer, enabling transmission at greater distances and over longer periods of time^5^. This definition has been challenged and may very well be an over-simplification and it may be precarious to rigidly differentiate the two categories^3,6,7^.

Previously, other coronaviruses have been shown to disperse via aerosols and ventilation, and have been determined to cause HVAC (heating, ventilation, air conditioning) associated and nosocomial infections as well as extensive hospital outbreaks^8–13^. In recent studies, extensive environmental contamination of SARS-CoV-2 in hospital settings has been demonstrated, and viral RNA has been found both in air samples and in samples from air vent openings in isolation rooms^14–18^. Also, the potential for the aerosol transmission route of SARS-CoV-2 is supported by other recent studies^17,19–21^. The increased risk for infection in indoor environments, as well as superspreading events, could be explained by airborne transmission^22–26^. In this context it is therefore vital to understand the amount of SARS-CoV-2 in confined spaces and the distances at which virus can be passively dispersed. Hospital rooms where COVID-19 patients are treated are obviously venues in which airborne transmission is both of great importance to understand, as well as a suitable environment to study this phenomenon. In this study from a COVID-19 infectious disease ward at Uppsala University Hospital, Sweden, we investigated if SARS-CoV-2 RNA could be detected in and near air vent openings in isolation rooms and in filters in the central ventilation system situated on the eighth (top) floor of the hospital building. As RNA was detected at substantial distances from patient areas, fluid sample collections were performed in an attempt to determine the potential infective ability of SARS-CoV-2 detected in the systems. Our findings may suggest both airborne dispersal of SARS-CoV-2 and possible long-distance dissemination of SARS-CoV-2 via ventilation air flow.

## Materials and methods

### Sampling strategy

**Figure 1:**
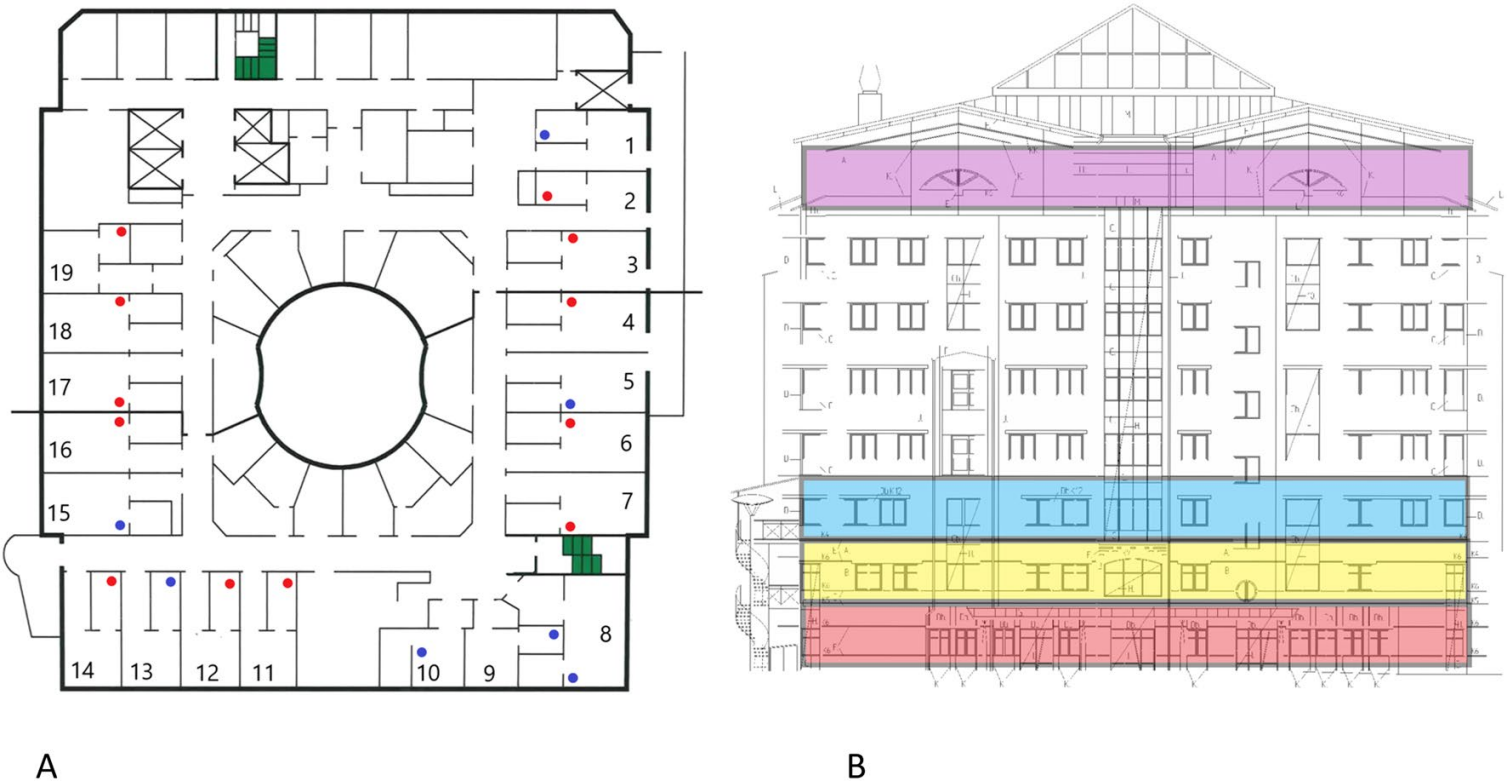
(**A**) Overview of the 19 investigated COVID-19 ward rooms (ward 1). Dots indicate approximate placing of ceiling vent openings. Red dots indicate openings that where SARS-CoV-2 RNA was detected in at least one of two samplings, blue dots openings negative in both samplings. (**B**) Lateral view of the hospital building. Ward levels: red; COVID-19 outpatient clinic, yellow and blue; COVID-19 wards 1 and 2, with 19 rooms each, purple; eighth floor with central ventilation fans and HEPA filters. Individual ceiling vent openings were investigated on the second-floor ward (yellow) seen in (**A**).

Sampling was performed on separate occasions during April and May 2020. In the first two occasions, 17 and 28 April, surfaces of exit vent openings in all 19 patient rooms in ward 1 (Fig. 1a) were swabbed as described below. When repeated on April 28, the internal surfaces of the central ventilation ducts, on the top floor were also swabbed and filter sections removed, as described further below. Due to the detection of SARS-CoV-2 RNA in the ventilation system (see “Results”), a further sample collection was performed using fluid traps, both at the terminal end of the ducts prior to the exhaust filters (at the same area where swabs were taken on April 28) as well as under the ceiling vent openings in the ward rooms (ward 1, see Fig. 1b), in an attempt to determine the infective ability of any collected virus.

### Swab samples

Surfaces were swabbed using sterile nylon flocked swabs (Copan eSwab, Copan Italia SpA, Italy) moistened in sterile viral transport medium (VTM), containing Hank’s balanced salt solution (Gibco, UK) supplemented with 2% fetal bovine serum (Gibco, USA), 100 µg/ml Gentamicin, and 0.5 µg/ml Amphotericin B^27^. Round ceiling vent openings were swabbed around the inside of the entire opening (circumference ca 25 cm). Swabs were placed in tubes containing 750 µl viral transport medium and stored at 4 °C until analysis within 24–72 h. Sampling was performed on April 17 and 28, 2020. Indoor relative air humidity and temperature were 30–31% and 20–21 °C, respectively.

### Filter samples

Exit ventilations from each of the eight stories in the investigated hospital building, (Fig. 1b), lead to separate HEPA filter systems, located on the eighth (top) floor. Consequently, we could identify ducts and exhaust filters collecting air from individual floors not merging airflows. We chose to examine exhaust filters from three floors in the building that had been specifically designated for COVID-19 patients; two COVID-19 wards and a COVID-19 out-patient clinic. In addition, we examined exhaust filters from one story with personnel areas and a cafeteria, as a negative control. The distance between the COVID-19 wards and the exhaust filters and inspection hatches was 49, 53 and 56 m respectively for each COVID-19 ward (Table 1). The four stories located between the COVID-19 wards and the central ventilation in the top of the building (Fig. 1b) only sporadically harbored COVID-19 patients and were therefore not investigated. Adjacent inspection hatches upstream from (prior to) the HEPA filters were opened, and internal 30 × 30 cm surfaces swabbed as described above. Furthermore, one (out of six) 60 × 60 cm laminate F7 HEPA filter sections was removed from each system (filtering air from one ward or floor) and three filter samples (3 × 3 cm) were randomly cut out of the filters using sterilized scissors, placed in vials containing 2.5 ml of viral transport medium (described above), and stored at 4 °C until analysis within 72 h. The removed filters had been routinely replaced one month prior to collection.

**Table 1:** rRT-PCR analysis of samples from filters and swabs in the ventilation system at the 8th floor, top level of the hospital building.

Corresponding floor	Exit airflow from ward (m^3^/s)	Approximate distance from ward to top floor filters (m)	Sample	PCR results (Ct value)	
				N gene	E gene
**Top floor air vent samples**					
Covid-19 outpatient clinic	2.45	56	Ventilation shaft swab	Negative	Negative
			Air vent filter sample 1	37.13	37.30
			Air vent filter sample 2	Negative	Negative
			Air vent filter sample 3	38.79	36.96
			Cell medium in petri dish	Negative	Negative
Covid-19 ward 1	2.27	53	Ventilation shaft swab 1	Negative	Negative
			Ventilation shaft swab 2	Negative	Negative
			Air vent filter sample 1	36.86	34.91
			Air vent filter sample 2	36.31	34.87
			Air vent filter sample 3	35.32	35.41
			Cell medium in petri dish	Negative	33.00
Covid-19 ward 2	2.55	49	Ventilation shaft swab	Negative	Negative
			Air vent filter sample 1	37.42	38.70
			Air vent filter sample 2	35.72	33.85
			Air vent filter sample 3	36.72	36.08
			Cell medium in petri dish	35.32	33.16
Ground level non-patient care area	3.48	60	Air vent filter sample × 3 (negative control)	Negative	Negative

Samples not exhibiting fluorescence above threshold level after 45 PCR cycles are labeled “negative”.

Ct: cycle threshold, N gene: SARS-CoV-2 Nucleocapsid gene, E gene: SARS-CoV-2 Membrane Small Envelope gene.

### Fluid samples

Fluid sample collection was performed near air entrances (ward rooms) and exits (directly prior to exhaust filters) in the ventilation system by placing open, 10 cm diameter petri dishes with 10 ml of DMEM (Dulbecco’s Modified Eagle’s medium (cell medium); Gibco) diluted 1 to 5 with autoclaved water, suspended 15 cm below ceiling vent openings (in ward rooms) for 24 h, or placed within central vent ducts via inspection hatches for 3 h. DMEM was diluted to ensure appropriate salt balance for the cells and no osmotic effect on the virus after evaporation of water during the collection process. We used DMEM instead of water only to be able to add the whole volume of sample onto cells without a dilution effect of the cell medium. These points were chosen in an attempt to determine if virus found entering and/or exiting the ventilation ducts retained infective ability, in response to the PCR results from vent opening and exhaust filters. The suspended petri dishes in the ward rooms were placed within what we expected to be the normal air flow to ventilation ducts, as well as placing the dishes as far from the patients as possible, in order to avoid contamination by coughing induced droplets or other patient or personnel activity in the rooms. An open petri dish containing cell medium was exposed to air in the biosafety level (BSL)-2 area of the laboratory for 24 h and used as a negative control, along with non-exposed DMEM and viral transport medium. DMEM and VTM spiked with synthetic oligonucleotides (gBlocks, IDT, Belgium) based on N and E gene sequences with introduced 5 base pair deletions were used as positive controls (Suppl. Table 1). DMEM exposed to air in 19 ward rooms were combined to three pools. Pooling was performed when we could establish that a large number of rooms were occupied by non-contagious patients (seven rooms), patients with suspected COVID-19 but not confirmed (five rooms) and only six rooms were occupied by contagious COVID-19 patients (May 13, 2020) (Suppl. Table 1). Due to evaporation during collection, the final concentration of DMEM in the petri dishes after collection was equivalent to undiluted cell medium. The entire pooled volume ranging from 5 to 10 ml was subsequently applied to Vero E6 cells in T25 flasks and incubated up to 13 days. Samples were subsequently collected and subject to rRT-PCR. Petri dishes with 10 ml DMEM exposed to air outside of patient areas for 24 h were used as negative control.

### Ward conditions

All exit vent openings in the ward rooms are situated in the ceiling and are approximately 3 to 5 m from the head end of the beds (fresh air input openings are at 0 to 50 cm above floor level). Seven of the 19 openings are situated in adjacent washing rooms (see Fig. 1a) and are up to approximately 5 to 6 m from beds. Total air changes per hour (ACH) for each patient room varied between 1.5 and 2.6 in ward 1, and 2.1 to 2.7 in ward 2, between 2.8 and 3.2 in the outpatient clinic, (measured December 2017). Air flow in the central ventilation shafts, from each story, ranged between 2.27 and 3.48 m^3^/s (Table 1). Pressure differences in rooms in ward 1 varied, − 6 to − 8.1 Pa between corridor and anterooms and + 5.5 to + 18 Pa between anterooms and patient rooms (measured March 2020). Hence, the anterooms were under negative pressure compared to the adjacent ward corridor as well as patient rooms.

### RNA extraction and rRT-PCR

RNA was extracted using 280 μl of samples and QIAamp viral RNA kit (Qiagen, Hilden, Germany), according to manufacturer’s protocol. Portions of the SARS-CoV-2 nucleocapsid (N) and envelope small membrane protein (E) genes were amplified by rRT-PCR, using primers (Thermo Fisher Scientific, Waltham, MA, USA) previously described^28–30^ and the SuperScript III OneStep RT-PCR System with Platinum Taq DNA Polymerase kit (Invitrogen, Carlsbad, CA, USA). In brief, the two reaction mixtures (25 μl) contained 12.5 μl reaction buffer (a buffer containing 0.4 mM of each dNTP, 3.2 mM MgSO_4_), 1 μl of enzyme solution (SuperScript III RT/Platinum Taq Mix), 1.25 μl of probe primers solution (10 µM stock concentration) 3 μl magnesium sulfate (50 nM), and 7.25 μl of RNA. The cycling conditions were as follows: cDNA synthesis at 55 °C for 30 min (min) and 50 °C for 2 min followed by 45 cycles of denaturation at 95 °C for 15 s (s), extension at 57 °C for 30 s and collecting the fluorescence signal at 68 °C for 30 s. Target 1 (E gene) forward primer ACAGGTACGTTAATAGTTAATAGCGT; reverse primer TGTGTGCGTACTGCTGCAATAT; and probe 5′-FAM-ACACTAGCCATCCTTACTGCGCTTCG-TAMRA-3′. Target 2 (N gene) forward primer GGGGAACTTCTCCTGCTAGAAT; reverse primer CAGCTTGAGAGCAAAATGTCTG; and probe 5′-FAM-TTGCTGCTGCTTGACAGATT-TAMRA-3′. As positive controls, double stranded DNA fragments (gBlocks, IDT, Belgium) with a five-nucleotide deletion in the targeted part of the E (10^2^ copies/µl) and N (10^3^ copies/µl) gene were used. Positive control Ct vales were 31.67 ± 0.68 and 28.07 ± 2.66 respectively. All PCR products with a Ct value < 45 were confirmed by Sanger sequencing (Macrogen, the Netherlands). Negative controls (swabs) were performed on non-exposed VTM (Suppl. Table 1).

### Inoculation

Vero E6 cells (green monkey kidney cells (ATCC CRL-1586)) were seeded into T-25 flasks and grown in DMEM (Gibco, 41966) supplemented with 10% FBS (Gibco, USA) and 1 × Penicillin–Streptomycin (Sigma-Aldrich, PA333). The flasks were incubated (37 °C, 5% CO2) until cells confluency reached approximately 90%, after which the cell media was substituted with 9 ml of pooled samples supplemented with 2% FBS and 1 × Penicillin–Streptomycin. Potential cytopathic effect (CPE) was observed daily. Increase in viral load was determined by rRT-PCR, using 100 μl of supernatant from each T-25 flask at 0 (base line for comparison), 24 and 120 h post infection (hpi). rRT-PCR was also performed on DMEM exposed to air in a BSL-2 laboratory for 24 h (see section “Fluid samples”), non-exposed DMEM and DMEM spiked with SARS-CoV-2 synthetic oligonucleotide control sequence as negative and positive controls, respectively (Suppl. Table 1). Eleven days post inoculation, supernatants from the pooled samples (1 ml) were passed once into new flasks seeded with Vero E6 cells and containing 4 ml of cell media. Two days after the passage, samples were taken as described above for quantification by rRT-PCR. All procedures involving live virus were performed in a BSL-3 laboratory.

### Ethical approval

Approval for accessing patient information was granted from the Swedish Ethical Review Authority DNR 2020-01787. As this retrospective data collection was considered completely anonymized by the Ethics committee, the need for patient consent was waived by the Swedish Ethical Review Authority. The study was conducted according to good clinical and scientific practices and following the ethical principles of the Declaration of Helsinki.

## Results

### SARS-CoV-2 RNA detection from ward samples

In two consecutive surface sampling rounds, performed on April 17 and 28, 2020, both SARS-CoV-2 N and E gene RNA were detected in seven (36.8%) out of 19 vent openings, while 11 days later, four vents (21%) were positive for both genes. Ct values varied between 33.77 and 39.78 (Table 2) and sequences were confirmed by Sanger sequencing. All three pooled cell medium samples from patient room ceilings were positive for both genes; Ct values ranged between 33.41 and 36.64. Pool 1 (Fluid traps from 7 rooms occupied by confirmed COVID-19 patients) N gene 35.47 and E gene 36.4, Pool 2 (6 suspected COVID-19 patient rooms) N gene 33.41 and E gene 36.64; Pool 3, (5 suspected non-contagious patient rooms), N gene 34.07 and E gene 36.64). Despite the attempt to arrange the potentially most infective samples in pools 1 and 2, a retrospective overview of patient diagnostics revealed that PCR-positive patients occupied rooms generating samples in all three pools (Suppl. Table 2).

**Table 2:** Overview of results from the 19 investigated COVID-19 ward rooms (ward 1), including patient details regarding duration of symptoms, date when clinical sample was collected for PCR-diagnosis, PCR-result from clinical sample and ongoing oxygen therapies when ventilation samples were collected.

Room	Sample set	Patient details						Ventilation opening	
		Days since onset of symptoms	SARS-CoV-2 PCR			Respiratory support		PCR results (Ct value)	
			Patient sample date	PCR results (Ct value)		Current	Last 24 h	N gene	E gene
				N gene	E gene				
1	1	17	April 1, 2020	23.51	22.22	Oxygen	Oxygen	Negative	Negative
	2	8	April 21, 2020	19.14	18.64	HFNC	HFNC	Negative	Negative
2	1	11	April 15, 2020	31.68	32.55	Oxygen	Oxygen	35.33	33.77
	2	12	April 18, 2020	13.3	13.91	Oxygen	Oxygen	Negative	Negative
3*	1	10	April 12, 2020	16.89	16.86	Oxygen	Oxygen	37.94	37.90
		*Unoccupied*							
	2	16	April 15, 2020	25.47	25.43	None	Oxygen	38.82	37.76
		9	April 21, 2020	14.96	14.98	HFNC	Oxygen/HFNC		
4*	1	15	April 7, 2020	25.98	25.33	HFNC	HFNC	39.55	38.71
		*Unoccupied*							
	2	20	April 13, 2020	19.72	19.11	Oxygen	Oxygen/HFNC	Negative	Negative
		29	April 5, 2020	17.16	16.59	None	Oxygen		
5*	1	*Unoccupied*						Negative	Negative
		7	April 14, 2020	25.38	25.33	HFNC	HFNC		
	2	*Unoccupied*						Negative	Negative
		8	April 23, 2020	Negative	Negative	Oxygen	Oxygen		
6*	1	8	April 11, 2020	17.91	16.88	Oxygen	Oxygen	36.24	36.70
		20	March 31, 2020	25.18	24.1	HFNC	HFNC		
	2	5	April 25, 2020	Negative	Negative	None	None	Negative	36.78
		*Unoccupied*							
7*	1	7	April 16, 2020	22.84	22.5	None	None	39.28	Negative
		*Unoccupied*							
	2	*Unoccupied*						Negative	Negative
		16	April 22, 2020	32.19	Negative	Oxygen	Oxygen		
8*	1	*Unoccupied*						Negative	Negative
		1	April 17, 2020	Negative	Negative	None	None		
	2	*Unoccupied*						Negative	Negative
		15	April 21, 2020	16.09	15.99	None	Oxygen		
9	1	8	April 16, 2020	17.22	17.88	Oxygen	Oxygen	Negative	Negative
	2	12	April 24, 2020	23.76	23.7	None	None	Negative	Negative
10	1	20	April 5, 2020	21.95	21.57	HFNC	HFNC	Negative	Negative
	2	8	April 27, 2020	Negative	Negative	Oxygen	Oxygen	Negative	Negative
11	1	12	April 11, 2020	10.08	9.65	HFNC	Oxygen/HFNC	Negative	Negative
	2	*Unoccupied*						38.61	37.55
12	1	*Unoccupied*						39.77	38.95
	2	12	April 21, 2020	16.09	15.99	None	Oxygen	39.78	Negative
13	1	5	April 15, 2020	24.87	25	Oxygen	Oxygen/HFNC	Negative	Negative
	2	11	April 28, 2020	30.74	Negative	HFNC	HFNC	Negative	Negative
14	1	7	April 17, 2020	Negative	Negative	Oxygen	Oxygen	Negative	Negative
	2	8	April 26, 2020	23.55	22.04	HFNC	HFNC	38.75	38.45
15*	1	*Unoccupied*						Negative	Negative
		*Unoccupied*							
	2	15	April 20, 2020	14.95	14.83	Oxygen	Oxygen	Negative	Negative
		*Unoccupied*							
16*	1	13	April 13, 2020	15.95	15.47	Oxygen	Oxygen	37.26	36.14
		*Unoccupied*							
	2	23	April 14, 2020	17.91	17.58	HFNC	HFNC	Negative	Negative
		*Unoccupied*							
17*	1	18	April 16, 2020	31.03	36.18	None	None	Negative	Negative
		8	April 13, 2020	16.94	15.95	None	Oxygen		
	2	15	April 18, 2020	29.23	28.38	HFNC	HFNC	Negative	38.63
		30	April 8, 2020	25.31	25.44	Oxygen	Oxygen		
18*	1	*Unoccupied*						Negative	37.76
		18	April 6, 2020	19.02	17.62	Oxygen	Oxygen		
	2	*Unoccupied*						Negative	Negative
		*Unoccupied*							
19	1	14	April 6, 2020	14.28	13.58	Oxygen	Oxygen	37.56	35.28
	2	19	April 18, 2020	17.16	15.87	HFNC	HFNC	36.78	35.31

Rooms marked with an * can accommodate two patients, and thus patient data is supplied for two patients for each sample occasion. Sample set 1: April 17, 2020. Sample set 2: April 28, 2020. Samples not exhibiting fluorescence above threshold level after 45 PCR cycles are labeled “negative”.

No O_2_: No ongoing patient oxygen therapy, O_2_: conventional nasal cannula or mask, HFNC: High Flow Nasal Cannula, Ct: cycle threshold, N gene: SARS-CoV-2 Nucleocapsid gene, E gene: SARS-CoV-2 Membrane Small Envelope gene.

### SARS-CoV-2 RNA detection in central ventilation samples

Samples extracted from the main exhaust filters, located on the eighth (top) floor of the investigated hospital building (Fig. 1b), from each separate ventilation system for the three investigated COVID-19 wards were positive for both genes in eight (88.9%) out of nine samples (Table 2). Swabs taken from internal surfaces of three central ventilation channels at the top floor were all negative (Ct values > 45) (Table 1). Petri dishes containing cell medium, placed in inspection hatches in the central ventilation system prior to the exhaust filters, were found to contain SARS-CoV-2 RNA (both N and E genes) in one (33.3%) out of three specimens from ward 2 (Ct values 35.32 and 33.16 for N and E genes respectively), while one (33.3%) of the three specimens from ward 1 contained only the E gene (Ct value 33.00) (Table 1).

### Infectivity in Vero E6 cells

No significant CPE nor decrease in rRT-PCR Ct values were seen compared to baseline values (see “Results” above for Ct values) after 24 or 120 hpi on Vero E6 cells from samples retrieved from ward vent openings or central ventilation ducts or filters.

## Discussion

Several aspects during the COVID-19 pandemic support the risk of aerosol transmission of SARS-CoV-2. First, mounting evidence for pre- and asymptomatic transmission, where the spread of droplets through coughing and sneezing cannot be a major factor, must raise questions about aerosol transmission^31^. Second, aerosols generated by speech could theoretically contain enough SARS-CoV-2 virus particles to support transmission, and these aerosols can remain airborne for up to ten minutes^20^. In addition, coronaviruses can be emitted in aerosols through normal breathing^32^. Third, field studies in hospital wards have detected SARS-CoV-2 RNA both in vent openings and in the air^14–17^. These findings are not unexpected seeing as similar observations have been made for both SARS and Middle East Respiratory Syndrome (MERS)^8,33,34^.

In this study, we found SARS-CoV-2 RNA in vent openings in ward rooms harboring COVID-19 patients. Viral RNA was also detected in fluid placed in open dishes suspended below vent openings. Similar levels of viral RNA were detected in exhaust filters and open petri dishes with cell medium at least 44 to 56 m from the three investigated COVID-19 wards. Only a small fraction of each filter was analyzed implying that a large number of particles emanating from COVID-19 wards can disperse to greater distances than can be explained by droplet transmission routes. In previous studies, the effect of ventilation has not shown any obvious impact on the risk for spread of droplet-transmitted diseases, probably since droplets are more governed by gravity^35^. Furthermore, the ventilation system in the investigated hospital building has a relatively low air flow; between 1.7 and 3 total air changes per hour (ACH) for each room, depending on room volumes. The recommendation for airborne infection isolation rooms is 12 ACH in most guidelines^35^. Notably, the relative air humidity in the investigated environment was low, between 30 and 31%. Low air humidity has recently been suggested to increase the risk of airborne SARS-CoV-2 dispersal^36,37^.

We initiated this study by performing rRT-PCR on numerous surface and filter samples. Detection of SARS-CoV-2 as well as other coronavirus RNA in ventilation openings has been reported before^10,15,38^. However, the detection of viral RNA in the exhaust filters over 50 m from patient care areas was unexpected. In response to these findings, we found it vital to rapidly address the question of infective ability in order to determine the immediate risk of infection for uninfected patients, personnel working in the investigated wards and service personnel that might be exposed while working with the ventilation systems. We therefore employed the ad hoc methods described above in an attempt to determine the infective ability of the samples. We are aware that there are several potential limitations to the employed sampling methods in fluid traps; the likelihood of viral particles being deposited in fluids by gravity, the length of time the viral particles retain infective ability, concentration and increased osmolarity of the cell medium by evaporation as well as pH increase due to oxygen exposure during sampling. We have not determined whether the detected RNA could be from viral particles that have been inactivated by antibodies, seeing as a majority of the patients admitted to at least one of the wards were in later phases of COVID-19 disease at both collection dates (Table 2), and may have likely developed an immune response. Even though we could not determine infective capability of virus collected in cell medium, we repeatedly detected SARS-CoV-2 RNA using this method. The placement of the petri dishes, either just below the ceiling in ward rooms or at distances around at least 50 m from patients in central vent ducts indicates that dispersal by means other than larger droplets must occur, since larger droplets are considered to precipitate by gravity within one or two meters from a source^5^. Although RNA could be detected in samples from ward rooms and central ventilation ducts, no infectivity was seen after inoculating samples on susceptible cells. This collection method was adopted in order to rapidly address the question as to what threat the RNA findings may infer in a clinical setting. Several explanations for these results may be identified. First, the Ct values are close to the detection limit, indicating that there were few viral copies in theses samples. Also, many of the admitted patients at this time point (later than other samplings in this study) were in late phases of COVID-19 or cleared of infection. We chose to report this as we could detect SARS-CoV-2 RNA in these samples, and that droplets do not appear to be a plausible explanation for these findings as droplets could unlikely follow a ballistic pathway from patient into the petri dished at 2.5 m height, and in all three pools. It is important to continue to develop effective sampling methods in order to determine infective ability of SARS-CoV-2 as well as differentiating between patients in early and late phases of disease. Since we are aware of these technical limitations, we have recommended service personnel to take adequate protective measures while working with the ventilation systems as we cannot definitively repudiate the risk of infection from contaminated air.

Ongoing oxygenation therapies, such as High Flow Nasal Cannula (HFNC) oxygenation, in each room did not apparently correlate to detection, or Ct values, of SARS-CoV-2 RNA in vent openings (Table 2). This raises the question if the risk for airborne transmission should be considered in more situations than during potentially aerosol generating procedures such as HFNC^6^. This is further corroborated by the studies on aerosols generated when speaking and breathing^20,32^. Results differed in ward rooms between the two samplings of vent openings, which could be due to varying disease progression for the occupying patients. Some vent openings were positive for both N and E genes despite the rooms having been evacuated and routinely cleaned (Table 2). This suggests that detection also could result from viral shedding by previous patients and calls for further studies on how long SARS-CoV-2 RNA can be detected in the environment, with the accompanying risk for transmission via fomites. Alternatively, detection of viral RNA in the ventilation systems could arise from such activities as handling bed linens or cleaning which may disturb viral particles from textiles or surfaces and displace them into the air, and that these virions have dried and been rendered inactive. On the other hand, RNA deterioration after inactivation could limit the extent of this source of RNA found in HVAC systems.

In this study we could not demonstrate infectious capability of the virus, when inoculated on Vero E6 cells, from samples in either vent openings, exhaust filters or by collection directly in cell medium. This is likely due to the pathogens rapidly drying in the vents or inadequate amounts of virus collected near vent openings or in front of exhaust filters. Also, collection directly in cell medium does not appear to have been performed previously and these results should be interpreted cautiously. Furthermore, admitted patients in the ward were between day 5 and 23 after symptom onset (Table 2). There is accumulating evidence that COVID-19 contagiousness peaks shortly prior to symptom onset^2,31,39^. This implies that the patients in this study may be in a less contagious phase of COVID-19 disease, which is consistent with the findings that SARS-CoV-2 infectivity appears to be low eight days after symptom onset^39,40^. Nevertheless, during dispersal from a patient to ventilation, and over considerable distances, the virus may still retain infective capability. RNA was also detected in containers placed at ceiling level, demonstrating that viral particles were airborne during these specific periods, at not only deposited on fomites over longer, uncertain duration. We speculate that the risk of infection by exposure to ventilation system air is presumably very low, due to dilution of viral load and drying. Nevertheless, the apparent capability of the virus to be transported in air, as we present here, should raise concerns for the risk of infection in smaller, confined spaces in close proximity to contagious patients, i.e. all air in patients rooms, intensive care units, etc. during care for COVID-19 patients^41^. This may be even more important concerning patients in earlier phases of disease, in which contagiousness may be high. This includes both symptomatic and asymptomatic SARS-CoV-2 infected persons in any confined space, such as homes, public transportation, restaurants, etc. The presented findings indicate airborne dissemination of SARS-CoV-2, especially considering the distance SARS-CoV-2 RNA was dispersed. However, further investigations, preferably discriminating between patients in early and later phases of SARS-CoV-2 disease as well as direct sampling of expiratory air from COVID-19 patients will be needed to resolve this question.

## Conclusions

Detection of coronavirus RNA, including SARS-CoV-2, in hospital and other ventilation systems has been reported, as well as nosocomial and HVAC associated outbreaks^8–13^. In particular, MERS coronavirus, closely related to SARS-CoV-2, has caused major hospital associated outbreaks^9–12^. Also, growing concern about aerosol transmission of SARS-CoV-2 has recently been ventilated^42^. Here we present further evidence for SARS-CoV-2 ability to disperse from patients to ward vent openings as well as detection of viral RNA in ventilation exhaust filters located at least 50 m from patient room vent openings. Although we could not conclude that the viral samples in this collection retained infective ability, the distance at which we detected RNA suggests that there may be a risk for airborne dissemination and transmission, especially at much closer distances to contagious persons in confined spaces, both in and outside hospital environments. We therefore find it reasonable to take precautionary measures against airborne transmission and that further investigations are necessary.

## Supplementary information

Supplementary Information.

## Abbreviations

ACH: Air changes per hour
CoV: Coronavirus
COVID-19: Coronavirus infectious disease 2019
Ct: Cycle threshold
HFNC: High flow nasal cannula
Hpi: Hour post infection
HVAC: Heating Ventilation Air-condition
MERS: Middle eastern respiratory syndrome
RNA: Ribonucleic acid
rRT-PCR: Real time reverse transcriptase polymerase chain reaction
SARS: Severe acute respiratory syndrome
