# Supplementary material for: Long-distance airborne dispersal of SARS-CoV-2 in COVID-19 wards
Source: Sci Rep. 2020 Nov 11;10:19589. doi: 10.1038/s41598-020-76442-2 (PMC7659316; doi:10.1038/s41598-020-76442-2)
Supplement: Supplementary file 1 — Supplementary Information. [file 41598_2020_76442_MOESM1_ESM.docx]

**Supplement Nissen et al.**

Long-distance airborne dispersal of SARS-CoV-2 in COVID-19 wards

Karolina Nissen MD^1^, Janina Krambrich^2^, Dario Akaberi^2^, Tove Hoffman^2^, Jiaxin Ling PhD^2^, Åke Lundkvist PhD^2^, Lennart Svensson PhD^3,4^, Erik Salaneck MD PhD^1^

^1^Dept of Medical Sciences, Uppsala University, Uppsala, Sweden

^2^Dept of Medical Biochemistry and Microbiology, Uppsala University, Uppsala, Sweden

^3^Dept of Molecular Medicine and Virology, Linköping University, Linköping, Sweden

^4^Div of Infectious Diseases, Dept of Medicine Karolinska Institute, Solna, Sweden

1. **Supplementary methods**


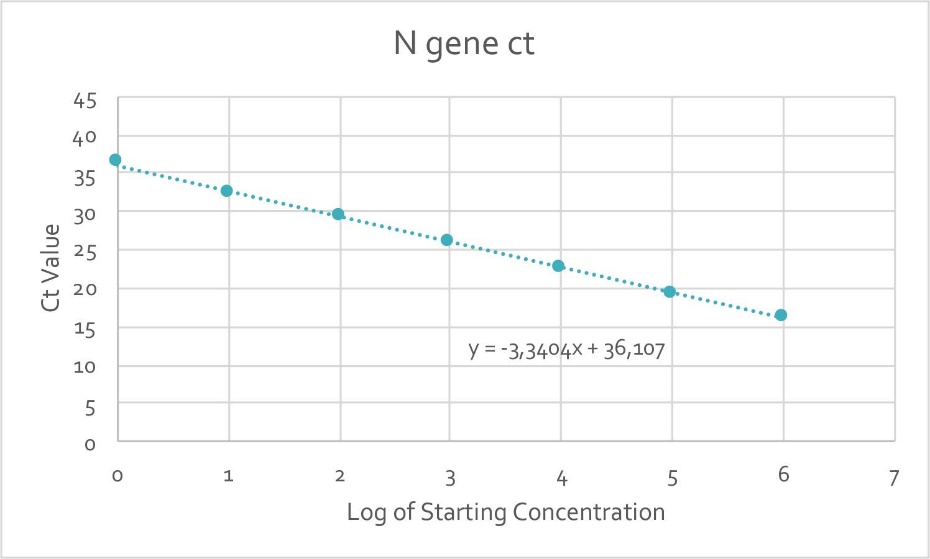

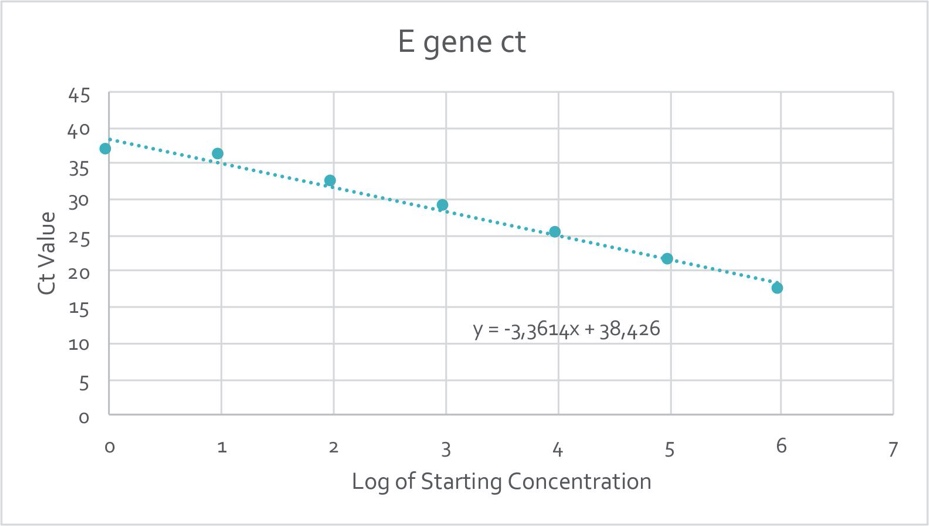


**Supplementary figure 1**. Standard curves of positive controls (synthetic gene fragments, gBLOCKs) used for rRT-PCRs targeting the E and N gene regions of SARS-CoV-2. Initial quantity of gBLOCKs used were 10^0^ to 10^6^ copies/µl. Limit of detection (LOD) and efficiency (E) calculated by 10-fold serial dilution in triplicate: LOD_E-gene_ 10 copies/µl and LOD_N gene_ 10 copies/µl; E_E gene_ = 101.5% and E_N gene_ = 103.1%. The coefficient of determination (R^2^) were: R^2^_E gene_ = 0.987, R^2^_N gene_ = 0.998. Linear dynamic range (LDR) - the concentration range over which the analytes were reliably determined were: LDR_E gene_ = 10^6^ to 10^-1^ copies/µl and LDR_N gene_ = 10^6^ to 10^-1^ copies/µl. The specificity was determined by amplicon size, visualized by gel electrophoresis, and Sanger sequencing.

| **Positive controls in matrix (DMEM and Viral Transport Medium)** | | | | | |
| --- | --- | --- | --- | --- | --- |
| **Sample (gBLOCK synthetic control)** | **Used copy number** | | **Ct value according to standard curve** | **Ct value after extraction** | **Difference in ct value** |
| E gene in DMEM | 4 x 10^6^ | | 16.233 | 17.12±0.011 | 0.887 |
| E gene in VTM | 4 x 10^6^ | | 16.233 | 16.91 ±0.108 | 0.677 |
| N gene in DMEM | 4 x 10^6^ | | 14.191 | 16.53±0.078 | 2.339 |
| N gene in VTM | 4 x 10^6^ | | 14.191 | 15.75±0.094 | 1.559 |
| **Negative controls (matrix only)** | | | | | |
| **Sample (matrix)** | **Ct value after extraction** |  |  |  |  |
| DMEM | >45 |  |  |  |  |
| VTM | >45 |  |  |  |  |

**Supplementary Methods table 1.** Positive controls (synthetic oligonucleotides, gBLOCKs) in the matrices Dulbecco’s Modified Eagle’s Medium (DMEM) and Viral Transport Medium (VTM) and negative controls consisting of only matrix (DMEM or VTM). All controls were run in triplicate.

1. **Supplementary Table**

| **Pool 1, room numbers** | **Symptom onset** | **PCR diagnosis** | **Note** |
| --- | --- | --- | --- |
| 2 | April 15 | April 18 |  |
| 4 (2 patients) | April 25 and April 14 | May 4 and April 19 |  |
| 6 (2 patients) | April 29 and April 30 | May 5 and May 6 |  |
| 12 (unoccupied) |  |  | Discharged May 5 |
| 15 | April 25 | May 3 and May 20 |  |
| 17 (2 patients) | May 7 and April 26 | May 10 and April 29 |  |
| 19 |  | Negative CoV-PCR | Suspected May 12 |

| **Pool 2, room numbers** | **Symptom onset** | **PCR diagnosis** | **Note** |
| --- | --- | --- | --- |
| 3 | April 25 | April 27 |  |
| 9 | May 6 | May 12 |  |
| 10 | Not documented | Negative CoV-PCR | CT thorax* |
| 13 | May 1 | May 7 |  |
| 18 (2 patients) | April 18 and April 9 | April 25 and April 16 |  |

*Patient with radiological (computer tomography thorax) picture concurrent with COVID-19

| **Pool 3, room numbers** | **Symptom onset** | **PCR diagnosis** | **Note** |
| --- | --- | --- | --- |
| 1 | NA | Negative CoV-PCR |  |
| 5 | NA | Negative CoV-PCR |  |
| 7 | NA | Negative CoV-PCR |  |
| 8 (unoccupied) |  |  |  |
| 11 | April 25 | Negative CoV-PCR |  |
| 14 | March 30 | April 5 |  |
| 16 | April 30 | April 30 |  |

**Supplementary table 2.** Patient data for pooling of fluid air traps in patient rooms collected May 12-13, 2020. Fluids were exposed for 24 hours, 15 cm below ceiling level in all patient rooms. When two patients occupied one room, dates for symptom onset and PCR diagnosis are listed respectively for each patient (rooms 4, 6, 17, and 18). Pools were organized aiming at concentrating positive or highly suspected positive SARS-CoV-2 patients to pool 1, moderately suspected or clinically improving patients to pool 2, and negative or suspected negative patients to pool 3. Ct values of rRT-PCR reactions performed on each pool can be seen in table 1 in the article.
